# Predicting 2-year survival in stage I-III non-small cell lung cancer: the development and validation of a scoring system from an Australian cohort

**DOI:** 10.1186/s13014-022-02050-1

**Published:** 2022-04-13

**Authors:** Natalie Si-Yi Lee, Jesmin Shafiq, Matthew Field, Caroline Fiddler, Suganthy Varadarajan, Senthilkumar Gandhidasan, Eric Hau, Shalini Kavita Vinod

**Affiliations:** 1 South Western Sydney Clinical School, Faculty of Medicine, University of New South Wales, Sydney, Australia; 2 Ingham Institute for Applied Medical Research, Liverpool, NSW Australia; 3 Illawarra Cancer Care Centre, Wollongong, NSW Australia; 4 Blacktown Cancer and Haematology Centre, Blacktown Hospital, Blacktown, NSW Australia; 5 Crown Princess Mary Cancer Centre, Westmead Hospital, Westmead, NSW Australia; 6 University of Sydney, Sydney, NSW Australia; 7 Cancer Therapy Centre, Liverpool Hospital, Locked Bag 7103, Liverpool BC, NSW 1871 Australia

**Keywords:** Non-small cell lung cancer, Radiotherapy, Survival prediction

## Abstract

**Background:**

There are limited data on survival prediction models in contemporary inoperable non-small cell lung cancer (NSCLC) patients. The objective of this study was to develop and validate a survival prediction model in a cohort of inoperable stage I-III NSCLC patients treated with radiotherapy.

**Methods:**

Data from inoperable stage I-III NSCLC patients diagnosed from 1/1/2016 to 31/12/2017 were collected from three radiation oncology clinics. Patient, tumour and treatment-related variables were selected for model inclusion using univariate and multivariate analysis. Cox proportional hazards regression was used to develop a 2-year overall survival prediction model, the South West Sydney Model (SWSM) in one clinic (n = 117) and validated in the other clinics (n = 144). Model performance, assessed internally and on one independent dataset, was expressed as Harrell’s concordance index (c-index).

**Results:**

The SWSM contained five variables: Eastern Cooperative Oncology Group performance status, diffusing capacity of the lung for carbon monoxide, histological diagnosis, tumour lobe and equivalent dose in 2 Gy fractions. The SWSM yielded a c-index of 0.70 on internal validation and 0.72 on external validation. Survival probability could be stratified into three groups using a risk score derived from the model.

**Conclusions:**

A 2-year survival model with good discrimination was developed. The model included tumour lobe as a novel variable and has the potential to guide treatment decisions. Further validation is needed in a larger patient cohort.

## Background

Lung cancer is the leading cause of cancer incidence and mortality globally, of which non-small cell lung cancer (NSCLC) comprises 85% [1]. In Australia, the majority of NSCLC patients are diagnosed at stage I-III, with guidelines recommending curative radiotherapy in for those who are medically inoperable or refuse surgery [2].

Over the last two decades, while there has been an increase in the use of curative treatment [3], significant patterns of radiotherapy underutilisation persist across Australia and internationally [4]. Despite optimal radiotherapy rates being 61–76% of NSCLC patients, in practice this falls to 42–43% [5] due to a multitude of reasons including patient comorbidity and clinician bias. One strategy for addressing such variation is the development of a survival prediction model that integrates individual, medical and environmental factors unaccounted for by guidelines that commonly influence treatment decisions. This would have the potential to objectively evaluate treatment benefits in individual patients to facilitate shared decision-making, tailor patient management and optimise outcomes [6].

At present, the tumour, node, and metastasis (TNM) classification is considered gold standard for NSCLC prognostication. However stage alone is a poor predictor of overall survival, accounting for less than half of prognostic variance [7]. NSCLC patients within the same anatomic stratification are inherently heterogenous, with actual prognosis depending on a complex interplay of patient, tumour and treatment characteristics [8]. To accurately predict NSCLC survival beyond TNM stage and clinical judgement alone [9], quantitative survival prediction models that can be applied to specific patient profiles must account for a range of predictive factors, reflect current practice and demonstrate higher concordance than existing prognostication methods.

While several models have been published, none have demonstrated superior performance, applicability or global utility [11]. There is considerable discordance in the factors included in prognostic tools, with a systematic review discussing incomplete coverage of established predictors and the incorporation of variables that are difficult to measure as key shortcomings of published models [10]. Additionally, the discriminatory accuracies of existing tools have generally been insufficient to justify deviation from conventional staging systems [11, 12]. Furthermore, with the development of newer radiotherapy protocols, targeted therapies and immunotherapy, earlier studies fail to capture contemporary approaches to NSCLC and are no longer clinically relevant [13]. There is a need for prediction models that incorporate comprehensive data from cohorts treated with modern radiotherapy techniques, and that encompass emerging factors such as mutation and programmed cell death-ligand-1 (PD- L1) status [14].

The primary aim of this study was to develop and validate a 2-year survival prediction model in a contemporary cohort of stage I–III NSCLC patients treated with radiotherapy. Secondary aims were to compare model survival predictions to those predicted by TNM stage and validate published survival prediction models in a comparable cohort of patients.

## Methods

### Population

This retrospective cohort study included patients diagnosed with inoperable stage I-III NSCLC between January 2016 and December 2017 at three Australian radiotherapy treatment institutions. Tumour staging was performed based on multidisciplinary team recommendations. All patients treated radically were staged using positron emission tomography-computed tomography (PET-CT), with confirmation using endobronchial ultrasound and biopsy in instances of uncertainty or where there was the potential to influence management. Patients who received radiotherapy alone or chemoradiotherapy (concurrent or sequential) were eligible for inclusion. Patients treated surgically or for recurrent disease were excluded. The development cohort comprised patients from South Western Sydney Local Health District (SWSLHD). The validation cohort included patients from Blacktown Cancer and Haematology Centre and Illawarra Cancer Care Centre (BICC).

### Data

Retrospective data were retrieved through automated and manual extraction methods using the electronic medical record systems MOSAIQ (Elekta AB, Stockholm, Sweden), ARIA (Varian Medical Systems, Palo Alto, CA) and Cerner Powerchart (Cerner Corp, North Kansas City, MO). Gross tumour volume (GTV) data were obtained from radiotherapy planning systems for patients who received radiotherapy.

Data were collected for all available predictive variables for survival as identified from a prior literature review. Patient-related variables included: age at diagnosis, sex, current smoking status, pack years smoked, weight loss, pre-treatment pulmonary function (percent predicted values for forced expiratory volume in 1 s (FEV1) and diffusing capacity of the lung for carbon monoxide (DLCO)), Eastern Cooperative Oncology Group (ECOG) performance status [15] and comorbidities as defined by the Simplified Comorbidity Score (SCS) [16].

Tumour-related variables were: TNM stage according to the International Association for the Study of Lung Cancer (IASLC) 8th edition [17], histology, tumour grade, GTV, tumour location, mutation status (epidermal growth factor receptor (*EGFR*), anaplastic lymphoma kinase (*ALK*) and V-raf murine sarcoma viral oncogene homolog B (*BRAF*)) and PD-L1 status [18]. GTV was defined as the sum of the GTV primary and GTV lymph nodes.

Treatment-related variables included radiotherapy technique, radiotherapy treatment duration, equivalent dose in 2 Gy fractions (EQD_2_) and use of chemotherapy. Radiotherapy across the three cohorts included both conventional and stereotactic ablative body radiotherapy (SABR), with radiotherapy technique classified as conformal, intensity-modulated radiotherapy (IMRT) and volumetric modulated arc therapy (VMAT).

The primary outcome of overall survival was recorded as patient status at 31/12/2019 with all follow-up data obtained before this study end date. Survival time was defined as the period from the start of radiation therapy until date of death or until 31/12/2019 for living patients.

### Statistical analysis

The Kaplan–Meier method was used to predict survival in the study population. Variables missing > 50% of data were excluded from univariate and multivariate analysis. In the development cohort, univariate Cox proportional hazards regression was used to evaluate the predictive value of variables, with those demonstrating an association with overall survival (p < 0.20) considered for inclusion in multivariate analysis. Backward stepwise regression was applied to select the variables retained in the final multivariate model [19]. Model fit was evaluated using the Hosmer–Lemeshow goodness of fit test.

A scoring system for the South West Sydney Model (SWSM) was generated using the logarithm of the odds ratio (OR) to allocate points for each variable. Risk groups were defined according to total score quartiles in the development cohort. Kaplan–Meier curves were generated and log-rank test used to evaluate significant survival differences between subgroups. The model was applied to the SWSLHD cohort to assess internal validity, with discrimination estimated using Harrell’s concordance index (c-index) [20]. To assess the impact of missing DLCO data on model performance, validation was performed twice, initially with missing data excluded and again with simple mean imputation. Model calibration was assessed graphically by plotting observed survival probabilities against predicted probabilities and calculating the calibration slope and intercept for the development and validation cohorts [21]. External validation was performed by applying the SWSM to the BICC cohort.

The performance of the multivariate model was compared with predictions based on TNM staging alone. In addition, an existing prediction model was externally validated in the development and validation cohorts and compared to the SWSM. The discrimination of the model was also assessed using Harrell’s c-index.

Statistical analyses were performed using IBM SPSS Statistics, version 26.0 (IBM Corp, Armonk, NY), Matlab, version 9.3 (MathWorks, Natick, MA) and Python, version 3.6 (Python Software Foundation, Wilmington, DE). Ethics approval was obtained from the SWSLHD Human Research Ethics Committee.

## Results

The patient, tumour and treatment characteristics of each study site are summarised in Table 1. There were a total of 261 patients included in the study. At the study end date, 47.5% of patients were alive. In the development cohort, mutation status was unknown in 65.0% and PD-L1 status in 85.5% of patients and these variables were excluded from analysis.

**Table 1:** Characteristics of the development and validation datasets

Characteristic	Development cohort (n = 117)	Validation cohort (n = 144)	p-value^†^
	n (%)	n (%)	
Sex			0.1
Male	79 (67.5)	83 (57.6)	
Female	38 (32.5)	61 (42.4)	
Age (years)			0.31
Median (range)	73 (34–90)	74 (45–91)	
ECOG performance status			0.025
0	55 (47.0)	44 (30.6)	
1	35 (29.9)	61 (42.4)	
≥ 2	27 (23.1)	36 (25.0)	
Unknown	0 (0)	3 (2.1)	
SCS			0.16
0–9	77 (65.8)	104 (72.2)	
10–20	40 (34.2)	37 (35.6)	
Unknown	0 (0)	3 (2.1)	
Smoking status			0.53
Current	38 (32.5)	37 (25.7)	
Past	71 (60.7)	93 (64.6)	
Never	7 (6.0)	10 (6.9)	
Unknown	1 (0.9)	4 (2.8)	
Pack years smoked			0.7
Median (range)	40 (0–150)	40 (0–150)	
Unknown	7 (6.0)	7 (4.9)	
Weight loss (%)			< 0.001
None	57 (48.7)	41 (28.5)	
≤ 10	29 (24.8)	51 (35.4)	
> 10	25 (21.4)	7 (4.9)	
Unknown	6 (5.1)	45 (31.3)	
FEV1 (%)			0.19
Median (range)	68 (25–121)	68 (30–133)	
Unknown	16 (13.7)	36 (25.0)	
DLCO (%)			0.5
Median (range)	60 (28–115)	58 (28–134)	
Unknown	24 (20.5)	84 (58.3)	
Overall stage			0.016
I	34 (29.1)	62 (43.1)	
II	22 (18.8)	13 (9.0)	
III	61 (52.1)	69 (47.9)	
T stage			0.086
T1	31 (26.5)	54 (37.5)	
T2	38 (32.5)	52 (36.1)	
T3	33 (28.2)	25 (17.4)	
T4	14 (12.0)	13 (9.0)	
Unknown	1 (0.9)	0 (0)	
N stage			0.065
N0	56 (47.9)	79 (54.9)	
N1	14 (12.0)	5 (3.5)	
N2	31 (26.5)	42 (29.2)	
N3	16 (13.7)	18 (12.5)	
Histological diagnosis			0.034
Squamous cell carcinoma	64 (54.7)	24 (16.7)	
Adenocarcinoma	37 (31.6)	45 (31.3)	
Other	16 (13.7)	69 (47.9)	
Tumour grade			0.11
< 3	36 (30.8)	24 (16.7)	
3	39 (33.3)	45 (31.3)	
Unknown	42 (35.9)	75 (52.1)	
Tumour laterality			0.23
Left	48 (41.0)	71 (49.3)	
Right	67 (57.3)	73 (50.7)	
Unknown	2 (1.7)	0 (0)	
Tumour lobe			0.76
Upper	71 (60.7)	86 (59.7)	
Middle/lower	44 (37.6)	57 (39.6)	
Unknown	2 (1.7)	1 (0.7)	
GTV (cc)			0.32
Median (range)	49.8 (0.7–1039.0)	23 (1.7–1065.0)	
Unknown	26 (22.2)	29 (20.1)	
Mutation status			0.28
*EGFR* positive	4 (3.4)	8 (5.6)	
*ALK* positive	1 (0.9)	2 (1.4)	
None	36 (30.8)	30 (20.8)	
Unknown	76 (65.0)	104 (72.2)	
PD-L1 status (%)			0.45
< 1	4 (3.4)	2 (1.4)	
1–49	8 (6.8)	4 (2.8)	
≥ 50	5 (4.3)	2 (1.4)	
Unknown	100 (85.5)	136 (94.4)	
Radiotherapy technique			< 0.001
Conformal	15 (12.8)	80 (55.6)	
IMRT	80 (68.4)	24 (16.7)	
VMAT	22 (18.8)	39 (27.1)	
Unknown	0 (0)	1 (0.7)	
Radiotherapy treatment duration (days)			0.37
Median (range)	26 (1–47)	22 (1–48)	
EQD_2_ (Gy)			0.12
Median (range)	60 (12–126)	60 (6.3–88)	
Chemotherapy use			0.18
Chemotherapy	45 (38.5)	44 (30.6)	
No chemotherapy	72 (61.5)	100 (69.4)	
Survival time (months)			0.011
Median (range)	17 (1–44)	22 (0–46)	
2-year status			0.92
Alive	56 (47.9)	68 (47.2)	
Dead	61 (52.1)	76 (52.8)	

ECOG = Eastern Cooperative Oncology Group; SCS = Simplified Comorbidity Score; FEV1 = forced expiratory volume in 1 s; DLCO = diffusing capacity of the lung for carbon monoxide; GTV = gross tumour volume; *EGFR* = epidermal growth factor receptor; *ALK* = anaplastic lymphoma kinase, PD-L1 = programmed death-ligand 1; IMRT = intensity-modulated radiotherapy; VMAT = volumetric modulated arc therapy; EQD_2_ = equivalent dose in 2 Gy fractions

^†^Comparison of development and validation cohort

By univariate analysis, the variables predictive of survival in the study population were ECOG performance status, DLCO, overall stage, histological diagnosis, tumour lobe, radiotherapy technique, radiotherapy treatment duration and EQD_2_ (Table 2). On multivariate analysis, the variables predictive of survival were ECOG performance status, DLCO, histological diagnosis, tumour lobe and EQD_2_ (Table 3). The Hosmer–Lemeshow test demonstrated good model fit using the selected predictors (p = 0.65). The scoring system for the SWSM is presented in Table 4.

**Table 2:** Survival predictors in the development cohort by univariate and multivariate Cox proportional hazards regression

Variable	2-year status		Univariate analysis		Multivariate analysis	
	Alive (n = 56)	Dead (n = 61)	OR (95% CI)	p-value	OR (95% CI)	p-value
Sex				0.96	NI^†^	–
Male	38	41	1.00 (Reference)			
Female	18	20	1.01 (0.59–1.73)			
Age (years)			1.01 (0.99–1.04)	0.41	NI	–
Median (SD)	71.4 (10.6)	75.0 (10.2)				
ECOG performance status				0.03		0.1
0	28	27	1.00 (Reference)		1.00 (Reference)	
1	21	14	0.81 (0.43–1.56)	0.54	0.65 (0.36–1.16)	0.14
≥ 2	7	20	1.87 (1.04–3.36)	0.036	1.27 (0.70–2.31)	0.43
SCS				0.68	NI	–
0–9	36	41	1.00 (Reference)			
10–20	20	20	1.12 (0.65–1.93)			
Smoking status				0.53	NI	–
Never	3	4	1.00 (Reference)			
Past	35	36	0.77 (0.27–2.18)	0.63		
Current	18	20	1.05 (0.36–3.08)	0.93		
Pack years smoked			1.00 (0.99–1.01)	0.9	NI	–
Median (SD)	40.0 (29.4)	43.6 (28.3)				
Weight loss (%)				0.22	NI	–
None	29	28	1.00 (Reference)			
≤ 10	15	14	1.00 (0.53–1.92)	0.98		
> 10	9	16	1.68 (0.90–3.11)	0.1		
FEV1 (%)			0.99 (0.98–1.01)	0.42	NI	–
Median (SD)	66.0 (21.3)	67.3 (18.7)				
DLCO (%)			0.99 (0.97–1.00)	0.078	0.99 (0.97–1.00)	0.046
Median (SD)	64.0 (23.0)	59.7 (15.9)				
Overall stage				0.09		0.37
I	20	14	1.00 (Reference)		1.00 (Reference)	
II	7	15	2.20 (1.06–4.59)	0.034	0.86 (0.38–1.96)	0.72
III	29	32	1.33 (0.71–2.52)	0.37	1.62 (0.99–2.66)	0.36
T stage				0.41	NI	–
T1	18	13	1.00 (Reference)			
T2	20	18	1.08 (0.53–2.21)	0.84		
T3	12	21	1.63 (0.81–3.29)	0.17		
T4	6	8	1.57 (0.65–3.79)	0.32		
N stage				0.44	NI	–
N0	31	25	1.00 (Reference)			
N1	5	9	1.87 (0.87–4.03)	0.11		
N2	14	17	1.29 (0.69–2.40)	0.42		
N3	6	10	1.25 (0.60–2.64)	0.55		
Histological diagnosis				0.025		0.078
Squamous cell carcinoma	25	39	1.00 (Reference)		1.00 (Reference)	
Adenocarcinoma	21	16	0.52 (0.29–0.94)	0.029	0.57 (0.34–0.95)	0.031
Other	10	6	0.36 (0.13–1.04)	0.058	0.62 (0.28–1.38)	0.24
Tumour grade				0.82	NI	–
< 3	17	19	1.00 (Reference)			
3	17	22	1.07 (0.58–1.99)			
Tumour laterality				0.72	NI	–
Left	26	22	1.00 (Reference)			
Right	30	37	1.10 (0.65–1.87)			
Tumour lobe				0.056		0.059
Upper	38	33	1.00 (Reference)		1.00 (Reference)	
Middle/lower	18	26	1.66 (0.98–2.80)		1.61 (0.98–2.62)	
GTV (cc)			1.00 (1.00–1.00)	0.88	NI	–
Median (SD)	21.6 (173.2)	78.7 (92.3)				
Radiotherapy technique				0.005		0.92
Conformal	4	11	1.00 (Reference)		1.00 (Reference)	
IMRT	38	42	0.42 (0.21–0.83)	0.012	0.95 (0.37–2.39)	0.9
VMAT	14	8	0.25 (0.10–0.62)	0.003	0.83 (0.29–2.39)	0.73
Radiotherapy treatment duration (days)			0.99 (0.97–1.00)	0.15	0.99 (0.96–1.00)	0.52
Median (SD)	26.7 (14.2)	21.0 (15.2)				
EQD_2_ (Gy)			0.97 (0.96–0.99)	< 0.001	0.98 (0.96–0.99)	< 0.001
Median (SD)	63 (16.6)	60 (18.6)				
Chemotherapy use				0.28	NI	–
Chemotherapy	22	23	1.00 (Reference)			
No chemotherapy	34	38	1.34 (0.79–2.27)			

OR = odds ratio; CI = confidence interval; SD = standard deviation; ECOG = Eastern Cooperative Oncology Group; SCS = Simplified Comorbidity Score; FEV1 = forced expiratory volume in 1 s; DLCO = diffusing capacity of carbon monoxide; GTV = gross tumour volume; IMRT = intensity-modulated radiotherapy; VMAT = volumetric modulated arc therapy; EQD_2_ = equivalent dose in 2 Gy fractions

^†^NI = Not included in multivariate analysis as p-value > 0.20 by univariate analysis

**Table 3:** Odds ratios of SWSM for overall survival by multivariate analysis

Variable	β coefficient	SE	OR	Log OR	95% CI	p-value
ECOG performance status						0.1
0			1.00 (Reference)	0.00 (Reference)		
1	− 0.43	0.3	0.65	− 0.19	0.36–1.16	0.14
≥ 2	0.24	0.31	1.28	0.11	0.70–2.31	0.43
DLCO	− 0.014	0.006	0.99	− 0.004	0.97–1.00	0.022
Histological diagnosis						0.078
Squamous cell carcinoma			1.00 (Reference)	0.00 (Reference)		
Adenocarcinoma	− 0.57	0.26	0.57	− 0.24	0.34–0.95	0.031
Other	− 0.48	0.41	0.62	− 0.21	0.28–1.38	0.24
Tumour lobe						0.059
Upper			1.00 (Reference)	0.00 (Reference)		
Middle/lower	0.47	0.25	1.61	0.21	0.98–2.62	
EQD_2_	− 0.025	0.006	0.98	− 0.009	0.96–0.99	< 0.001

SWSM = South West Sydney Model; SE = standard error; OR = odds ratio; CI = confidence interval; ECOG = Eastern Cooperative Oncology Group; DLCO = diffusing capacity of the lung for carbon monoxide; EQD_2=_ equivalent dose in 2 Gy fractions

**Table 4:** Scoring system for the SWSM

Variable	Points
ECOG performance status	
0	2
1	0
≥ 2	3
DLCO (%)	
> 132	0
118–132	1
102–117	2
87–101	3
72–86	4
56–71	5
41–55	6
26–40	7
< 26	8
Histological diagnosis	
Squamous cell carcinoma	3
Adenocarcinoma	0
Other	1
Tumour lobe	
Upper	0
Middle/lower	2
EQD_2_	
> 85	0
77–85	1
68–76	2
59–67	3
50–58	4
41–49	5
32–40	6
23–31	7
14–22	8
5–13	9
< 5	10
Risk group	
1	< 10
2	10–15
3	> 15

SWSM = South West Sydney Model; ECOG = Eastern Cooperative Oncology Group; DLCO = diffusing capacity of the lung for carbon monoxide; EQD_2_ = equivalent dose in 2 Gy fractions

**Fig. 1:**
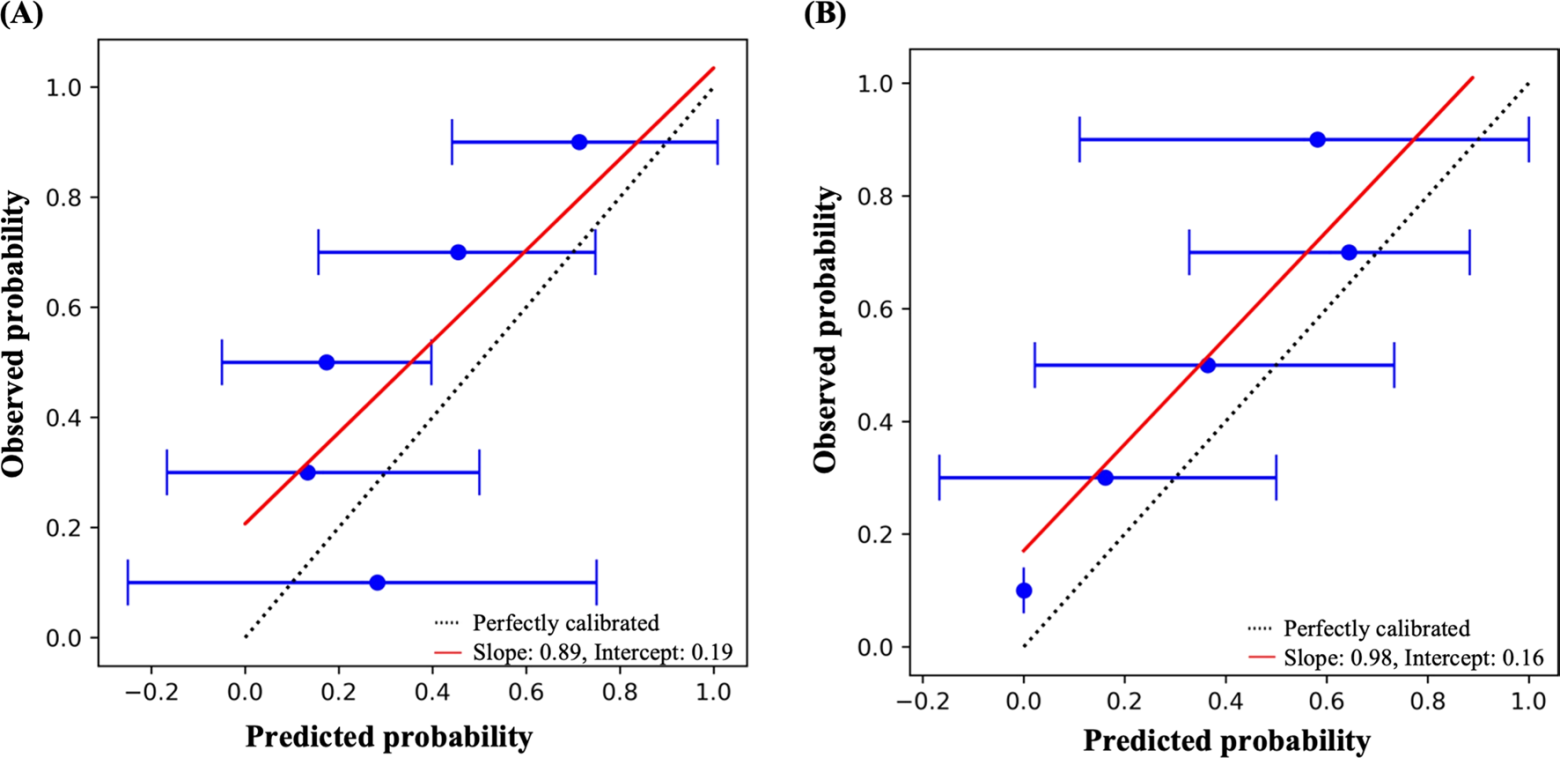
Calibration plots for the SWSM. SWSM calibration plots in the **A** development cohort (n = 117) and **B** validation cohort (n = 144)

Internal validation of the SWSM model demonstrated a c-index of 0.70, 95% CI [0.64, 0.75]. Discrimination on the external validation cohort resulted in a c-index of 0.72, 95% CI [0.68, 0.76] with imputation and 0.67, 95% CI [0.58, 0.74] with missing DLCO data excluded. Calibration of the SWSM yielded a calibration slope of 0.89, 95% CI [0.40, 1.34] and intercept of 0.19, 95% CI [0.01, 0.35] in the development cohort and a calibration slope of 0.98, 95% CI [0.77, 1.16] and intercept of 0.16, 95% CI [0.06, 0.26] in the validation cohort (Fig. 1).

**Fig. 2:**
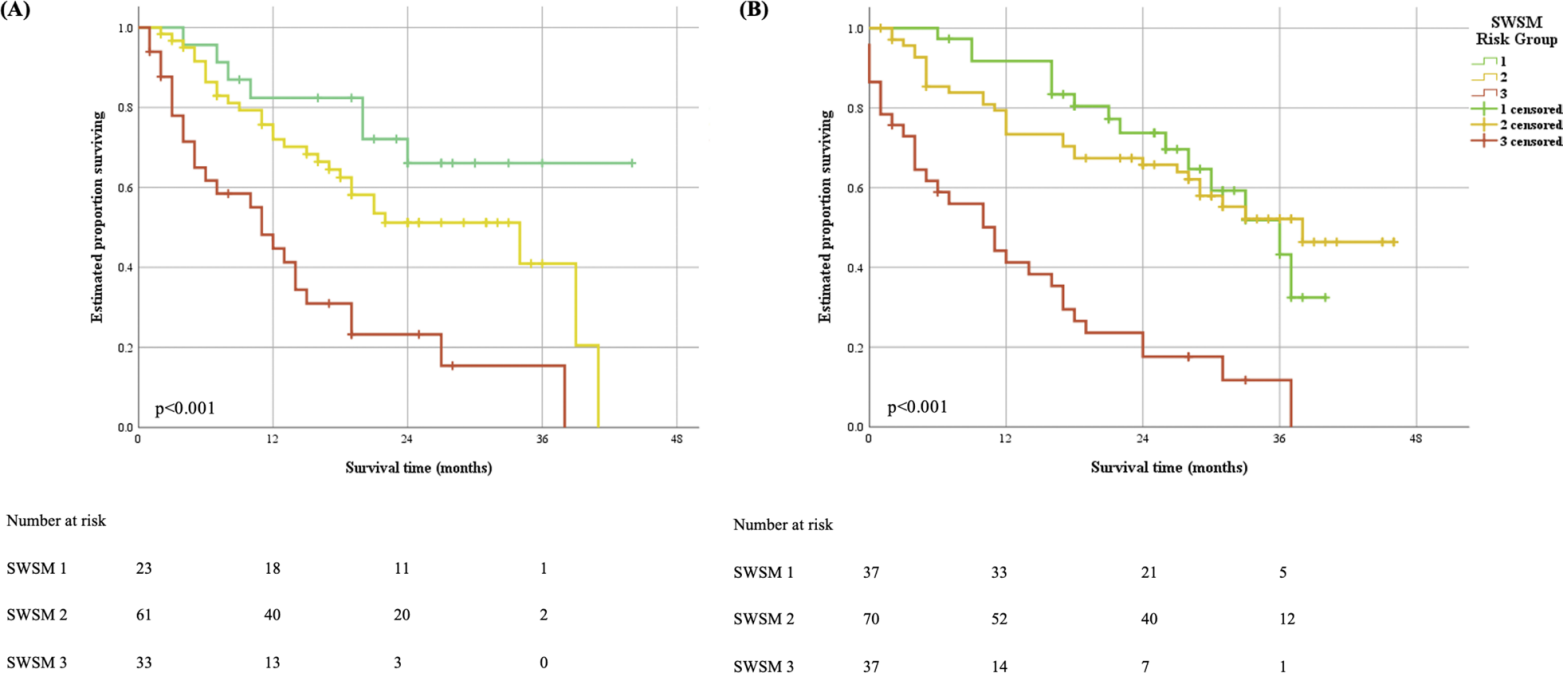
Survival curves according to SWSM risk group. Kaplan–Meier curves for cumulative survival of NSCLC patients by SWSM risk group in **A** development cohort (n = 117) and **B** validation cohort (n = 144). SWSM = South West Sydney Model

The formation of four risk groups in the SWSLHD cohort according to SWSM quartiles resulted in no differences between groups 2 and 3. Subsequently these groups were combined, producing a total of three risk groups. Kaplan–Meier survival curves by SWSM risk group in the development and validation cohorts are presented in Fig. 2. Log-rank test identified significant differences (p < 0.05) between risk groups in both development and validation cohorts. Using the SWSM, 2-year survival probability in the development cohort was 63.3% in group 1, 55.0% in group 2 and 20.0% in group 3.

The c-index of TNM staging alone for survival prediction was 0.53, 95% CI [0.48, 0.58] in the SWSLHD cohort and 0.59, 95% CI [0.55, 0.63] in the BICC cohort. An existing model from the MAASTRO clinic [13] was externally validated to give c-indexes of 0.62, 95% CI [0.56, 0.67] in the SWSLHD cohort and 0.66, 95% CI [0.61, 0.70]in the BICC cohort.

## Discussion

To our knowledge, this study is the first to develop and validate a survival prediction model in a cohort of inoperable but potentially curable stage I-III NSCLC patients irrespective of radiotherapy intent. Developing a survival prediction model in this cohort may impact on management decisions as although in theory all patients should be treated with curative radiotherapy, this does not happen in real world practice. The SWSM incorporates a combination of well-established and novel predictors using routinely collected data to predict survival in a radiotherapy cohort. Notably, our incorporation of EQD_2_ enables the SWSM to be applied as a predictive models with the potential to facilitate treatment decisions and radiotherapy planning.

When evaluating the predictive performance of a model, a c-index greater than 0.6 generally reflects helpful discrimination [22]. In the development cohort, the SWSM demonstrated good predictive performance, and achieved similar results on external validation in one independent cohort. There was minimal difference between the c-indexes obtained on external validation when missing data were excluded or imputed. Importantly, the SWSM maintained discrimination superior to survival predictions based on TNM staging alone. This is in accordance with published models whereby the addition of demographic and clinical covariates translated into more robust survival predictions [14, 23, 24].

In prediction models, calibration illustrates the association between predicted and observed outcomes. A slope of one and intercept of zero indicate perfect calibration [25]. While our model calibration plot showed good agreement between expected and actual survival probabilities, the slope and positive intercept are suggestive of survival underestimation. Validation in a larger population is required for the generation of a more precise calibration curve.

Several models predicting survival in inoperable NSCLC cohorts have been published [11, 14, 15], however none have examined the entire inoperable stage I-III radiotherapy cohort in a contemporary setting. The MAASTRO model is a prognostic tool for 2-year survival of stage I-III NSCLC patients treated with curative chemoradiotherapy. However, since its 2009 publication, staging classifications and radiotherapy techniques have been updated. The c-indexes obtained during external validation of the SWSM were higher than those obtained on validation of the MAASTRO model [13]. In addition, the advantages of our model include its reflection of current practice and potential to be applied to patients receiving curative or palliative therapy.

Similarly, models published more recently have been limited to patients receiving curative radiotherapy or with early or localised disease. The STEPS (sex, T stage, Staging EBUS, performance status, N stage) score was developed in the UK to predict 2-year risk of death in stage I-III NSCLC and also contained five variables [26]. However, only patient and tumour-related factors were retained in this multivariate model, precluding its use in treatment decision-making. Another model developed in Japan only included patient-related variables (age, performance status, body mass index and Charlson comorbidity index) to predict non-lung cancer death in an elderly cohort receiving definitive SABR [27]. In contrast to these models, we deliberately chose to include a potentially curable population of patients with stage I-III NSCLC who were managed heterogeneously in order to develop a prediction model which can help decision-making in the real world.

During the development of the SWSM, the predictive variables considered for model inclusion encompassed patient, tumour and treatment factors as supported by current evidence. The patient-related determinants most commonly included in NSCLC models are age, sex and performance status. While a poorer prognosis has been associated with older age [27] and male sex [8] overall findings have been inconclusive. In line with previous models, our study found no significant associations between age and sex on the survival outcomes of the study population [28, 29]. In contrast, performance status has been associated with NSCLC survival in patients receiving curative radiotherapy [23, 26, 28, 30] and was retained in our final model. However, a recognised limitation of performance status as a predictive variable is its subjectivity and inter-observer variability [27].

The only other patient-related variable identified as a survival predictor was pre-treatment DLCO. This has been demonstrated in a model involving NSCLC patients treated with SABR [30]. Insufficient lung function is a common reason for medical inoperability and frequently determines the suitability of treatment, with one study identifying pre-treatment DLCO as the pulmonary function measure most strongly associated with overall survival [31]. While DLCO has been reported to influence NSCLC survival in the surgical literature [32], few studies have analysed its influence on inoperable cohorts. The results of this study could be used to support further research exploring this association.

The tumour-related characteristics included in the SWSM were histological diagnosis and tumour lobe. Consistent with prior studies, non-squamous cell tumours demonstrated better survival probability than squamous cell carcinomas [17, 33]. The inclusion of tumour lobe in the SWSM is novel. A recent systematic review concluded that tumours located in the upper lobe conferred improved survival compared to those in the middle or lower lobes [34], consistent with our findings. The increased treatment toxicities from higher cardiac dose for middle lobe tumours and higher lung dose for lower lobe tumours may explain this result. Furthermore, the lower lobe has been associated with an increased proportion of non-adenocarcinoma tumours and a lower frequency of EGFR mutations, both of which are unfavourable survival characteristics [35]. However, at present, evidence supporting the significance of tumour location as an independent predictor of survival is less established.

The inclusion of the treatment variable EQD_2_ in the SWSM allows its application as a predictive rather than a prognostic model [36]. By including EQD_2_ in the prediction model, using the scoring in Table 4, one can calculate survival depending on dosage regimen in an individual patient and counsel patients accordingly about risks and benefits of treatment. Some patients who only derive a small survival benefit from more intensive radiotherapy may not wish to risk the toxicities of treatment [37]. Others may choose to undergo higher dose radiotherapy for any survival benefit no matter how small. The model attempts to provide some objectivity to aid decision-making rather than relying on clinician judgement alone. Few studies have considered EQD_2_ as a variable [23] as most have been developed in a specific population only receiving curative treatment [11, 37]. Furthermore, while the survival benefit of increasing radiotherapy dose has been demonstrated, its ability to improve quality of life is yet to be established [38].

There are limitations to this study. The SWSM was developed using single institution data with a relatively small sample size, although the sample size is similar to other studies [37, 39]. The model was developed on the population demographic of South West Sydney, which has a higher proportion of overseas-born individuals compared to Australia as a whole, hence this may impact generalisability. This was a retrospective study relying on information documented in medical records resulting in inevitable missing data. Information not routinely collected within oncology information systems such as blood parameters were not analysed. Furthermore, data on the staging procedures used for individual patients were not collected, although this is reflective of a clinic cohort of patients. Survival may also have been impacted on by treatment at relapse which was not accounted for in this study. However the greatest impact on survival is initial treatment, and our methodology is similar to other survival prediction modelling studies [13, 39].

The findings of this study have implications for further research. External validation studies should be conducted by applying the SWSM to larger datasets to confirm findings and assess model generalisability. The current model may potentially be improved by the addition of mutation and PD-L1 status. Unfortunately, the influence of these predictors was unable to be evaluated as data on these markers were not routinely collected in stage I-III NSCLC patients during the time period of the study. Likewise, global advances in laboratory biomarkers and genomic parameters [40, 41] have been recently highlighted and may transform future NSCLC prognostication, however at present lack systematic investigation. In addition, data were not collected on cardiac radiation dose, which has been identified as an independent risk factor for all-cause mortality after radiotherapy in locally advanced NSCLC [42, 43]. Finally, impact analysis studies evaluating the acceptability, cost-effectiveness and practicality of the SWSM is required prior to clinical implementation [44].

We plan to develop and validate a survival prediction model in patients with Stage I-III NSCLC patients undergoing radiotherapy in a larger cohort of patients with distributed learning across multiple centres using the AusCAT network [45]. The factors found to be significant in this work will be considered alongside newer variables. The ultimate aim is to develop a tool to support radiotherapy decision-making in NSCLC using objective parameters rather than subjective clinical judgment. This will facilitate shared decision-making between patients and clinicians and reduce variability in treatment recommendations between clinicians and between institutions.

## Conclusions

In conclusion, our study developed a survival prediction model in a real-world contemporary cohort of inoperable stage I–III NSCLC patients treated with radiotherapy. The SWSM utilises readily obtainable data and is convenient and simple to use by clinicians. The model exhibited good discrimination on both internal and external validation, and has the potential to guide treatment decisions. Further validation of this model is needed in a larger cohort of patients.

## Data Availability

The datasets used during the current study are available from the corresponding author on reasonable request.

## Abbreviations

NSCLC: Non-small cell lung cancer
TNM: Tumour, node, and metastasis
PD-L1: Programmed cell death-ligand-1
PET-CT: Positron emission tomography-computed tomography
SWSLHD: South Western Sydney Local Health District
BICC: Blacktown Cancer and Haematology Centre and Illawarra Cancer Care Centre
GTV: Gross tumour volume
FEV1: Forced expiratory volume in 1 s
DLCO: Diffusing capacity of the lung for carbon monoxide
ECOG: Eastern Cooperative Oncology Group
SCS: Simplified Comorbidity Score
IASLC: International Association for the Study of Lung Cancer
EGFR: Epidermal growth factor receptor
ALK: Anaplastic lymphoma kinase
BRAF: V-raf murine sarcoma viral oncogene homolog B
EQD2: Equivalent dose in 2 Gy fractions
SABR: Stereotactic ablative body radiotherapy
IMRT: Intensity-modulated radiotherapy
VMAT: Volumetric modulated arc therapy
SWSM: South West Sydney Model
OR: Odds ratio
C-index: Concordance index

## References

[1] Bray F (2018). Global cancer statistics 2018: GLOBOCAN estimates of incidence and mortality worldwide for 36 cancers in 185 countries. CA Cancer J Clin.

[2] Cancer Council Australia Lung Cancer Guidelines Working Party. Clinical practice guidelines for the treatment of lung cancer. 2020. https://wiki.cancer.org.au/australia/Guidelines:Lung_cancer. Accessed 20 June 2021.

[3] Koshy M (2015). Disparities in treatment of patients with inoperable stage I non-small cell lung cancer: a population-based analysis. J Thorac Oncol.

[4] Vinod SK (2015). International patterns of radiotherapy practice for non–small cell lung cancer. Semin Radiat Oncol.

[5] Vinod SK (2010). Underutilization of radiotherapy for lung cancer in New South Wales. Australia Cancer.

[6] Nguyen AD (2019). Radiotherapy patterns of care for stage I and II non-small cell lung cancer in Sydney, Australia. J Med Imaging Radiat Oncol.

[7] Detterbeck FC (2016). The IASLC Lung Cancer Staging Project: methodology and validation used in the development of proposals for revision of the stage classification of NSCLC in the forthcoming (eighth) edition of the TNM classification of lung cancer. J Thorac Oncol.

[8] Lin J (2015). A prognostic model to predict mortality among non-small-cell lung cancer patients in the U.S. military health system. J Thorac Oncol.

[9] Oberije C (2014). A prospective study comparing the predictions of doctors versus models for treatment outcome of lung cancer patients: a step toward individualized care and shared decision making. Radiother Oncol.

[10] Mahar AL (2015). Refining prognosis in lung cancer: a report on the quality and relevance of clinical prognostic tools. J Thorac Oncol.

[11] Bouwmeester W (2012). Reporting and methods in clinical prediction research: a systematic review. PLoS Med.

[12] Jochems A (2018). A prediction model for early death in non-small cell lung cancer patients following curative-intent chemoradiotherapy. Acta Oncol..

[13] Dehing-Oberije C (2009). Development and external validation of prognostic model for 2-year survival of non-small-cell lung cancer patients treated with chemoradiotherapy. Int J Radiat Oncol Biol Phys.

[14] Alexander M (2017). Lung cancer prognostic index: a risk score to predict overall survival after the diagnosis of non-small-cell lung cancer. Br J Cancer.

[15] Oken MM (1982). Toxicity and response criteria of the Eastern Cooperative Oncology Group. Am J Clin Oncol.

[16] Colinet B (2005). A new simplified comorbidity score as a prognostic factor in non-small-cell lung cancer patients: description and comparison with the Charlson's index. Br J Cancer.

[17] Chansky K (2017). The IASLC Lung Cancer Staging Project external validation of the revision of the TNM stage groupings in the eighth edition of the TNM classification of lung cancer. J Thorac Oncol.

[18] Davis AA, Patel VG (2019). The role of PD-L1 expression as a predictive biomarker: an analysis of all US Food and Drug Administration (FDA) approvals of immune checkpoint inhibitors. J Immunother Cancer.

[19] Dunkler D (2014). Augmented backward elimination: a pragmatic and purposeful way to develop statistical models. PLoS ONE.

[20] Harrell FE, Lee KL, Mark DB (1996). Multivariable prognostic models: issues in developing models, evaluating assumptions and adequacy, and measuring and reducing errors. Stat Med.

[21] Moons KGM (2015). Transparent Reporting of a multivariable prediction model for Individual Prognosis Or Diagnosis (TRIPOD): explanation and elaboration. Ann Intern Med.

[22] Alba AC (2017). Discrimination and calibration of clinical prediction models: users’ guides to the medical literature. JAMA.

[23] Oberije C (2015). A validated prediction model for overall survival from stage III non-small cell lung cancer: toward survival prediction for individual patients. Int J Radiat Oncol Biol Phys.

[24] Putila J, Remick SC, Guo NL (2011). Combining clinical, pathological, and demographic factors refines prognosis of lung cancer: a population-based study. PLoS ONE.

[25] Van Calster B (2019). Calibration: the Achilles heel of predictive analytics. BMC Med.

[26] Evison M (2021). Predicting the risk of disease recurrence and death following curative-intent radiotherapy for non-small cell lung cancer: the development and validation of two scoring systems from a large multicentre UK cohort. Clin Oncol.

[27] Hanazawa H (2021). Development and validation of a prognostic model for non-lung cancer death in elderly patients treated with stereotactic body radiotherapy for non-small cell lung cancer. J Radiat Res.

[28] Louie AV (2015). Predicting overall survival after stereotactic ablative radiation therapy in early-stage lung cancer: development and external validation of the Amsterdam Prognostic Model. Int J Radiat Oncol Biol Phys.

[29] Jochems A (2017). Developing and validating a survival prediction model for NSCLC patients through distributed learning across 3 countries. Int J Radiat Oncol Biol Phys.

[30] Kang J (2020). Predicting 5-year progression and survival outcomes for early stage non-small cell lung cancer treated with stereotactic ablative radiation therapy: development and validation of robust prognostic nomograms. Int J Radiat Oncol Biol Phys.

[31] Guckenberger M (2012). Is there a lower limit of pretreatment pulmonary function for safe and effective stereotactic body radiotherapy for early-stage non-small cell lung cancer?. J Thorac Oncol.

[32] Berry MF (2015). Impact of pulmonary function measurements on long-term survival after lobectomy for stage I non-small cell lung cancer. Ann Thorac Surg.

[33] Yang Y (2022). Development and validation of a prediction model using molecular marker for long-term survival in unresectable stage III non-small cell lung cancer treated with chemoradiotherapy. Thorac Cancer.

[34] Lee HW, Lee C-H, Park YS (2018). Location of stage I-III non-small cell lung cancer and survival rate: systematic review and meta-analysis. Thorac Cancer.

[35] Lee HW (2020). Poor prognosis of NSCLC located in lower lobe is partly mediated by lower frequency of EGFR mutations. Sci Rep.

[36] Clark GM (2008). Prognostic factors versus predictive factors: examples from a clinical trial of erlotinib. Mol Oncol.

[37] Defraene G (2020). Multifactorial risk factors for mortality after chemotherapy and radiotherapy for non-small cell lung cancer. Radiother Oncol.

[38] Lewis TS (2020). Palliative lung radiotherapy: higher dose leads to improved survival?. Clin Oncol.

[39] Dekker A (2014). Rapid learning in practice: a lung cancer survival decision support system in routine patient care data. Radiother Oncol.

[40] Guo M (2019). Prognostic value of delta inflammatory biomarker-based nomograms in patients with inoperable locally advanced NSCLC. Int Immunopharmacol.

[41] He R, Zuo S (2019). A robust 8-gene prognostic signature for early-stage non-small cell lung cancer. Front Oncol.

[42] Atkins KM (2019). Cardiac radiation dose, cardiac disease, and mortality in patients with lung cancer. J Am Coll Cardiol.

[43] Thor M (2020). Modeling the impact of cardiopulmonary irradiation on overall survival in NRG Oncology Trial RTOG 0617. Clin Can Res.

[44] Cowley LE (2019). Methodological standards for the development and evaluation of clinical prediction rules: a review of the literature. Diagn Progn Res.

[45] Field M (2021). Implementation of the Australian Computer-Assisted Theragnostics (AusCAT) network for radiation oncology data extraction, reporting and distributed learning. J Med Imaging Radiat Oncol.

